# Adaptation of peroxisome proliferator-activated receptor alpha to hibernation in bats

**DOI:** 10.1186/s12862-015-0373-6

**Published:** 2015-05-17

**Authors:** Yijie Han, Guantao Zheng, Tianxiao Yang, Shuyi Zhang, Dong Dong, Yi-Hsuan Pan

**Affiliations:** Laboratory of Molecular Ecology and Evolution, Institute for Advanced Studies in Multidisciplinary Science and Technology, East China Normal University, Shanghai, 200062 China; State Key Laboratory of Estuarine and Coastal Research, East China Normal University, Shanghai, 200062 China; Laboratory of Molecular Ecology and Evolution, Institute of Estuarine and Coastal Research, East China Normal University, Shanghai, 200062 China

**Keywords:** PPARα, Hibernation, Bats, Mammals, Molecular evolution, Gene regulation

## Abstract

**Background:**

Hibernation is a survival mechanism in the winter for some animals. Fat preserved instead of glucose produced is the primary fuel during winter hibernation of mammals. Many genes involved in lipid metabolism are regulated by the peroxisome proliferator-activated receptor alpha (PPARα). The role of PPARα in hibernation of mammals remains largely unknown. Using a multidisciplinary approach, we investigated whether PPARα is adapted to hibernation in bats.

**Results:**

Evolutionary analyses revealed that the ω value of *Ppar*α of the ancestral lineage of hibernating bats in both Yinpterochiroptera and Yangochiroptera was lower than that of non-hibernating bats in Yinpterochiroptera, suggesting that a higher selective pressure acts on *Ppar*α in hibernating bats. PPARα expression was found to be increased at both mRNA and protein levels in distantly related bats (*Rhinolophus ferrumequinum* and *Hipposideros armiger* in Yinpterochiroptera and *Myotis ricketti* in Yangochiroptera) during their torpid episodes. Transcription factors such as FOXL1, NFYA, NFYB, SP1, TBP, and ERG were bioinformatically determined to have a higher binding affinity to the potential regulatory regions of *Ppar*α in hibernating than in non-hibernating mammals. Genome-wide bioinformatic analyses of 64 mammalian species showed that PPARα has more potential target genes and higher binding affinity to these genes in hibernating than in non-hibernating mammals.

**Conclusions:**

We conclude that PPARα is adapted to hibernation in bats based on the observations that *Ppar*α has a more stringent functional constraint in the ancestral lineage of hibernating bats and a higher level of expression in hibernating than in non-hibernating bats. We also conclude that PPARα plays a very important role in hibernation as hibernators have more PPARα target genes than non-hibernators, and PPARα in hibernators has a higher binding affinity for its target genes than in non-hibernators.

**Electronic supplementary material:**

The online version of this article (doi:10.1186/s12862-015-0373-6) contains supplementary material, which is available to authorized users.

## Background

Hibernation is an adaptive strategy used by some animals to survive cold winter weather when food is scarce [1, 2]. Small mammalian hibernators (e.g., squirrels and bats) undergo a series of torpor-arousal cycles during hibernation, in which a torpor bout lasts for several days or weeks but an arousal bout lasts only several hours [1, 3]. During torpor, the metabolic rate (MR) of these mammals is reduced to 2-4 % of the euthermic metabolism, and their body temperature (T_b_) may fall just a few degrees above the ambient temperature; however, both their MR and T_b_ are rapidly restored to the euthermic levels upon arousal [2]. The body weight and fat mass of these hibernators are dramatically increased in the pre-hibernation season. The preserved fats are used as the primary energy source during hibernation [2, 4, 5].

Bats belong to the order Chiroptera in the mammalian clade Laurasiatheria [6]. They comprise almost 1-quarter of mammalian species and are the only flying mammals [7, 8]. Hibernating bats (e.g., some species in superfamilies Rhinolophoidea, Emballonuroidea, and Vespertilionoidea) are mostly distributed in subtropical or cold latitudes, whereas non-hibernating bats are mainly reside in warm subtropical or tropical latitudes [9]. Many hibernating bat species in genera *Myotis* and *Rhinolophus* are deep hibernators that stringently control their overall metabolism during torpor [9, 10]. Positive selection in the coding regions of some genes, such as *Leptin* and *Bssl*, is found in these bats, suggesting that lipid metabolism has undergone adaptive evolution in response to hibernation [11, 12].

Peroxisome proliferator-activated receptors (PPARs) are nuclear receptors that regulate the expression of many genes (e.g., *Leptin*, *Hmgcs1*, *Ucp1*, and *Pgc1*α) involved in lipid and glucose homeostasis [13, 14] and the development of obesity, diabetes, and hypertension in mammals [15]. Ligand-dependent transcriptional regulation by PPARs depends on the heterodimerization of PPARs with their coactivators (e.g., RXR). PPARs can also be activated directly in a ligand-independent manner by phosphorylation [16, 17]. 3 different subtypes (α, β, and γ) of PPARs have been identified. PPARα is activated upon energy deprivation and is highly expressed in the liver, heart, kidney, and adipose tissues [17, 18], while subtypes β and γ, expressed ubiquitously, are associated with cell migration and adipocyte differentiation, respectively [19].

The actions of PPARα are regulated by the fibroblast growth factor 21 (FGF21), and the PPARα-FGF21 signaling cascade has been shown to induce torpor in fasting mice [20]. PPARα is recently shown to shift fuel utilization from carbohydrate to lipid during torpor of arctic ground squirrel, *Urocitellus parryii* [21]. A positive selection at the glycine residue of codon 96 (96G) of *Ppar*α has been detected in members of super-clade Laurasiatheria (placentals) and suborders of Euarchontoglires (primates and rodents) [22]. In addition, increased expression of PPARγ and its co-activator PGC-1α is observed in *Spermophilus tridecemlineatus* squirrels during a cold-induced torpor [23]. The expression of these 2 proteins is also increased in many organs of torpid *Myotis lucifugus* bats [24].

Although differential expression of PPARs and their co-activators (e.g., RXR) has been investigated in some hibernating mammals during different phases of hibernation [21, 23-26], the evolution of PPARα in hibernating bats remains unknown. Since PPARα plays a significant role in metabolic regulation during torpor [27], we hypothesize that PPARα has evolved in bats in adaption to hibernation. To test this hypothesis, we studied the evolution of *Ppar*α in fifty-six species of mammals including twelve species of bats. We also compared mRNA and protein expression of *Ppar*α in hibernating bats between torpid and arousal states. The number of potential PPARα target genes and the binding affinity of PPARα to these genes in hibernating and non-hibernating bats and other mammalian species were also determined.

## Results and Discussion

### Higher Selection Pressure on *Ppar*α in Hibernating Bats

**Fig. 1:**
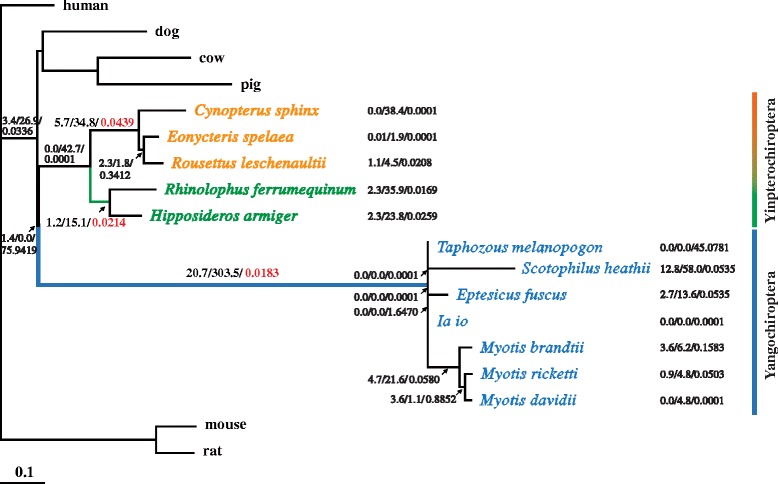
Species tree of *Ppar*α of bats. Non-hibernating bats in Yinpterochiroptera, hibernating bats in Yinpterochiroptera, and hibernating bats in Yangochiroptera are represented by orange, green, and blue colors, respectively. The N × *d*_*N*_/S × *d*_*S*_/ω value of each tree branch of bats is shown

To determine whether the coding region of *Ppar*α is positively selected in bats in response to hibernation, we conducted a branch model test on *Ppar*α from 9 hibernating and 3 non-hibernating species of bats by the maximum likelihood method using other mammalian species as an out-group (Fig 1 and Table 1). Although some of the branches were short and might lead to inaccurate estimation of ω (*d*_*N*_/*d*_*S*_), the ω value of the ancestral lineage of hibernating bats in both Yinpterochiroptera (Yin) and Yangochiroptera (Yang) was found to be lower than that of non-hibernating bats in Yin (Fig 1), suggesting a higher selective pressure acting on *Ppar*α in hibernating bats. A significant lower ω value was obtained by the 2-ratio model E (*P* = 0.008) when the ancestral branch of hibernating bats in Yang was labeled for comparison with other branches, indicating that *Ppar*α is much more conserved in this lineage (Table 1). It is conceivable that such conservation is required for the survival of hibernating bats, especially for those living in relatively low latitudes (see Additional file 3: Fig S1). No positive selection on any amino acid of *Ppar*α was found in the ancestral branches of hibernating bats in both Yin and Yang using the branch-site model A test (see Additional file 1: Table S1 and Additional file 2). There was also no positively selected site detected in these bats using the site model test (see Additional file 1: Table S2 and Additional file 2). However, *Ppar*α was found conserved across 56 mammalian species and experienced a higher selective pressure (ω = 0.057) in ancestral Chiroptera (see Additional file 1: Table S3, Additional file 2, and Additional file 3: Fig S2). These data suggest that *Ppar*α is conserved in evolution and support the previous postulation that heterothermy is an ancestral chiropteran trait [8, 12, 27]. A positive selection on codons 96 and 195 (96G and 195 V) of *Ppar*α was detected in these mammals by site model comparisons (M8-M7 model) (see Additional file 1: Table S4 and l file 2). This result is consistent with the positive selection on codon 96 (96G) of PPARα observed in super-clade Laurasiatheria (placentals) and suborders of Euarchontoglires (primates and rodents) [22].

**Table 1:** Branch model tests on *Ppar*α in bats

Model	np^a^	*ℓ* ^b^	ω_0_^c^	ω_labeled_^d^	Model compared	2Δ*ℓ*	P
A. One ratio: ω_0_	35	−5047.902	0.033	ω_0_			
B. One ratio: ω = 1 (fixed)	34	−6001.416	1.000	ω_0_	A and B	1907.029	0.000
C. Free ratio	67	−5016.255	—	—	A and C	63.294	0.001
D. Two ratios: ω_0_ = ω_H-Yan_, ω_H-Yin_	36	−5047.813	0.033	0.021	A and D	0.179	0.672
E. Two ratios: ω_0_ = ω_H-Yin_, ω_H-Yan_	36	−5044.408	0.037	0.018	A and E	6.988	0.008
F. Two ratios: ω_0_, ω_HN_	36	−5047.801	0.032	0.0042	A and F	0.203	0.653

^a^np: number of parameters.

^b^ *ℓ*: −Ln_likelihood ratio_.

^c^ω_0_: one ω ratio for all branches.

^d^ω_labeled_ are ω ratios for ancestral branches of Yinpterochiroptera hibernating bats (ω_H-Yin_), Yangopterochiropter hibernating bats (ω_H-Yan_), and non-hibernating bats (ω_HN_).

### Upregulation of *Ppar*α Expression in Torpid Bats

**Fig. 2:**
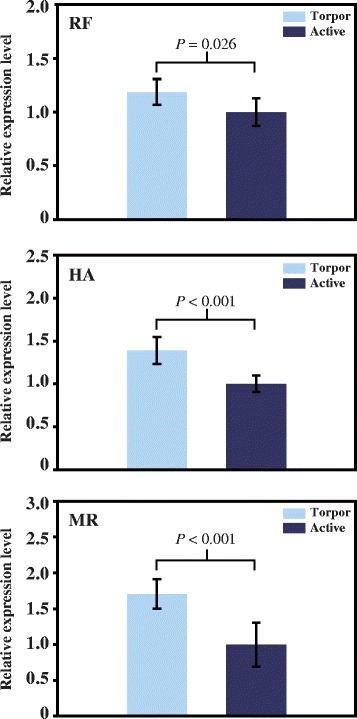
Expression levels of *Ppar*α mRNA determined by real-time RT PCR. RF, HA, and MR represent hibernating *R. ferrumequinum*, *H. armiger*, and *M. ricketti* bats, respectively. Relative mRNA levels in torpid and active states are indicated with light blue and dark blue colors, respectively. The expression level of *Ppar*α in active bats was set as 1.0. Data are presented as mean ± SD. A *P* value < 0.05 is considered statistically significant

We next investigated the possibility that bat *Ppar*α is adapted to hibernation at the level of transcription. The amount of *Ppar*α mRNA in the liver of torpid bats was compared to that of active bats, including the distantly related *Rhinolophus ferrumequinum* and *Hipposideros armiger* bats in Yin and *Myotis ricketti* bats in Yang (Fig 2). Results of quantitative PCR showed that *Ppar*α mRNA levels were significantly higher in torpid states than in active states and were increased by 1.19, 1.39, and 1.71 fold in torpid *R. ferrumequinum*, *H. armiger*, and *M. ricketti* bats, respectively (Fig 2). These results indicate that *Ppar*α in bats adapts to hibernation at the level of transcription.

The mechanisms for *Ppar*α upregulation in torpid bats remain to be investigated. During hibernation, lipid is the main fuel source, and a complex lipid signaling exists not only on the cell membrane but also in the nucleus [28]. Lipid signaling has a close relationship with the signaling pathways of numerous biological processes, such as inflammation, immunity, and glucose and amino acid metabolism [29-32]. PPARs are known to be activated by fatty acids and their derivatives [30, 31]. As some transcription factors may also activate *Ppar*α transcription, we used bioinformatic approaches to search for those that can bind to the 3 potential regulatory regions of *Ppar*α*,* including the region around the transcription start site (TSS_Around), the region upstream from TSS (TSS_Up), and the gene body (TSS_Body) (see Additional file 1: Table S5 and Additional file 2).

We found that transcription factors FOXL1, NFYA, NFYB, SP1, TBP, and ERG can bind to various regulatory regions of *Ppar*α in hibernating bats (FOXL1: TSS_Up 10 kb; NFYA, NFYB, and ERG: TSS_Body 7.5 kb; NFYB: TSS_Around 7.5 kb and TSS_Body 5 kb; SP1: TSS_Up 2.5 kb; TBP: TSS_Around 10 kb; ERG: TSS_Body 2.5 kb and 10 kb). In contrast, the NF-κB p65 subunit (RELA) and E2F1 were found to have the potential to bind to the regulatory regions of *Ppar*α in non-hibernating bats (RELA: TSS_Around 7.5 kb; E2F1: TSS_Body 10 kb). FOXL1 is a forkhead box transcription factor and is crucial for liver development and function. Knockdown of *Foxl1* in mice leads to defective intestinal glucose uptake [33]. NFYA, NFYB, and SP1 work cooperatively with PPARα to regulate the transcription of many lipogenic genes [34, 35]. TBP is a TATA box binding protein that interacts with PPARα to mediate gene transcription [36]. E2F1 regulates the expression of many genes involved in cell cycle control and proliferation [37]. RELA is a pleiotropic TF associated with the regulation of inflammation and immunity. Its activity is repressed by ligand-bound PPARs [37, 38].

It is known that E2F1 competes with histone deacetylase 1 (HDAC1) for binding to SP1 [39]. The activity of HDAC1 is significantly elevated in the skeletal muscle of *Spermophilus tridecemlineatus* ground squirrels during hibernation [40]. In these squirrels, the activation of NF-κB in skeletal muscle and intestine is positively linked to the antioxidant defense [41, 42]. NF-κB is also activated in the heart and muscle of torpid *Myotis lucifugus* bats [43]. These observations indicate that NF-κB and E2F1 are critical for hibernation. Since down regulation of PPARα depends on an intact signaling pathway of RELA [44] and E2F1 (an endogenous co-activator of NF-κB) [37], the potential of RELA and E2F1 to bind to the regulatory regions of *Ppar*α in non-hibernating bats suggests their roles in transcriptional repression of *Ppar*α. Similar binding preferences of FOXL1, NFYA, NFYB, SP1, TBP, ERG, RELA, and E2F1 on *Ppar*α were found in many other mammalian species (see Additional file 1: Table S5 and Additional file 2).

### Elevated Production of PPARα in Torpid Bats

**Fig. 3:**
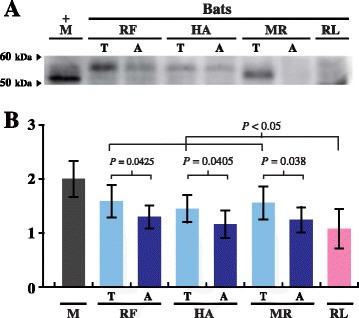
Expression levels of PPARα in bats determined by Western blotting. **(A)** Western blotting results of PPARα. **(B)** Relative levels of PPARα. The expression level of the PPARα protein in mice (M) was set as 2, and that of PPARα in each bat species was relative to it. RF, HA, and MR represent hibernating *R. ferrumequinum*, *H. armiger*, and *M. ricketti* bats, respectively. RL represents non-hibernating *R. leschenaultii* bats. T and A indicate bats in torpid and active states, respectively. Arrowheads in (A) indicate molecular weights (kDa) of protein markers. Relative levels of the PPARα protein in mice (gray), torpid (light blue) and active (dark blue) bats, and non-hibernating bats (pink) are shown in (B). Results are presented as mean ± SD. A *P* value < 0.05 is considered significant

To correlate the mRNA level of *Ppar*α with its protein level, we determined the amounts of PPARα protein in mice, hibernating *Rhinolophus ferrumequinum*, *Hipposideros armiger*, and *Myotis ricketti* bats, and non-hibernating *Rousettus leschenaultii* bats by Western blotting. Mouse PPARα (~55 kDa) protein was used as the positive control (Fig 3A). For each hibernating bat, the amount of PPARα in the torpid state (T) was compared to that in the active state (A). The levels of PPARα were found to be increased by 1.22, 1.24, and 1.25 fold in torpid *R. ferrumequinum*, *H. armiger*, and *M. ricketti* bats, respectively (*P* < 0.05) (Fig 3B). These results were consistent with the elevated expression of *Ppar*α mRNA in these bats during hibernation (Fig 2).

Because the expression of PPARα was upregulated in distantly related hibernating bats and its expression level was higher in torpid bats than in active and non-hibernating bats (Fig 3), these results strongly suggest the adaptation of bat PPARα to hibernation. The sizes of PPARα detected in samples from *R. ferrumequinum* and *H. armiger* bats were slightly higher than expected (Fig 3A). This discrepancy may be due to variations in post-translational modifications (PTMs) of PPARα as described previously [45]. The expression of PPARα in mice was found to be higher than in all the hibernating and nonhibernating bats examined (Fig 3), suggesting a potential hibernation capability of mice. This possibility is supported by the adenosine-induced torpid mice recently established by Swaop et al. [46].

In addition to using fat as the main energy source, bats maintain their blood glucose at 2–3 mM and catabolize amino acids to generate ketone bodies for energy supply during torpor [27]. PPARs are known to be involved in lipid metabolism and other cellular processes, such as insulin sensitivity, amino acid homeostasis, adaptation to starvation, and inflammation [31]. Overexpression of PPARα activates nearly every gene associated with fatty acid catabolism [31]. Furthermore, *Ppar*α*-*null mice exhibit increased glucose utilization, diminished fatty acid oxidation [15], and elevated expressions of many genes (e.g., arginase, phenylalanine hydroxylase, and spermidine synthetase) involved in amino acid metabolism [47]. Since hibernating mammals progressively suppress glucose utilization and use fat as the primary fuel [2], it is conceivable that bats produce sufficient amounts of PPARα to meet their metabolic requirements during torpor. Previous studies indicated that PPARα homeostasis is regulated by heterodimerization, recruitment of cofactors, post-translational modifications, and micro-RNAs (e.g., miR-519d) [48, 49]. Such regulation and the possible cross-talk among the various transcription factors observed in this study agree with the notion that transcriptional or translational reprogramming of genes occurs during mammalian hibernation [40].

### Variation of amino acid sequences of PPARα in bats

To investigate the adaptation of PPARα to hibernation, we compared the amino acid sequences of PPARα from 9 hibernating and 3 non-hibernating species of bats. The human PPARα sequence was used as the template. In the aligned region (amino acid positions 87 to 446), 316 amino acid sites were identical (87.78 %) and 44 amino acid sites were variable (see Additional file 3: Fig S3).

5 positions including V90, Y155, I195, T200, and I382 of PPARα were conserved in hibernating bats but were different or divergent among non-hibernating bats. 38 amino acid sites of PPARα were conserved in non-hibernating bats but were different among hibernating bats. Missense mutations at V90 and Y155 of PPARα had been reported in carcinomas of the large intestine and liver, respectively [50], and missense mutation at G96 had been observed in malignant melanoma [50]. The amino acid site 155 is located in the zinc-finger motif (C4 degenerated type, CX_2_CX_13_CX_2_CX_14–15_ CX_5_CX_9_CX_2_C) of PPARα (see Additional file 3: Fig S3). These results suggest the important role of these conserved amino acid sites in PPARα. Taken together, these data agree with the conservation and functional constraint of PPARα toward evolution (Fig 1 and Additional file 3: Fig S3).

### Potential PPARα Target genes

**Fig. 4:**
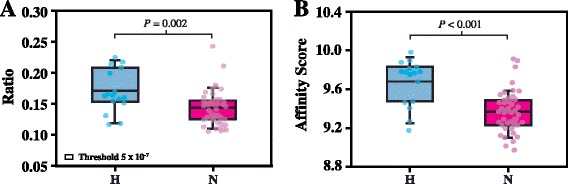
Bioinformatic analyses of PPARα in mammals. The y-axis in **(A)** represents the ratios of the number of PPARα potential target genes to the number of all genes of different animals. The small open rectangle indicates the threshold setting (5 x 10^−7^) for the matrix scan. The scores in **(B)** indicate the binding affinity of PPARα to its target genes in different animals. Blue and pink boxes indicate data calculated from 16 hibernating species (H) and 48 non-hibernating species (N) of mammals. Each dot represents the mean value obtained from each mammalian species. The box plot shows the median, 25/75 percentiles (box), and 10/90 percentiles (bars). A *P* value < 0.05 is considered significant

Genome-wide bioinformatic analyses were performed to estimate the mean ratio of the number of PPARα potential target genes to the number of all annotated genes (see Additional file 1: Table S6) in bats and to calculate the mean affinity score of PPARα to its target genes. 4 species of hibernating bats *Myotis brandtii*, *Myotis davidii*, *Myotis lucifugus*, and *Eptesicus fuscus* were found to have a higher mean target gene ratio than non-hibernating *Pteropus vampyrus* bats (see Additional file 3: Fig S4A), and the mean affinity score of PPARα to its target genes of these hibernating bats was higher than that of non-hibernating bats, *Pteropus vampyrus* and *Pteropus alecto* (see Additional file 3: Fig S4B). These results imply that mammalian hibernators have more PPARα target genes than non-hibernators and that PPARα has a higher binding affinity to its target genes in hibernators than in non-hibernators. To test these possibilities, the mean ratio of the number of PPARα potential target genes to the number of all genes in each of the 16 hibernating species and 48 non-hibernating species of mammals was determined; the mean affinity score of PPARα to its target genes in each of these mammalian species was also calculated. Results showed that the mean target gene ratio of the hibernators was significantly higher than that of the non-hibernators at a *P* value threshold of 5 x 10^−7^ (Fig 4A). This result was consistent across different settings of threshold *P* values (see Additional file 3: Fig S5 and Additional file 4). The mean affinity score of PPARα to its target genes in the hibernators was 9.68 ± 0.24, which is significantly higher than the score 9.37 ± 0.21 of the non-hibernators (Fig 4B and Additional file 1: Table S6). These data showed that hibernating mammals have more PPARα target genes and that PPARα has a higher affinity to its target genes in hibernating than in non-hibernating mammals.

To investigate phylogenetic inferences [51], we constructed a phylogenetic tree of 64 mammals (see Additional file 3: Fig S6A). The correlation between mammalian hibernation and the target gene ratio, or affinity score of PPARα, was analyzed by 2 different phylogenetic comparative methods, including the quantitative genetic threshold model proposed by Felsenstein [52] and the phylogenetic ANOVA proposed by Garland et al. [53] (see Additional file 2). With both approaches, we found that the correlation between hibernation and affinity score of PPARα was significant (95 % confidence interval 0.198 to 0.261; *P* < 0.002; see Additional file 1: Table S7). The correlation between hibernation and target gene ratio of PPARα was also significant (*P* value threshold 5 x 10^−7^) (see Additional file 1: Table S7 and Additional file 3: Fig S6B). However, the correlation coefficients were substantially decreased if the phylogeny of these mammals was considered. Therefore, phylogenesis is an important factor in the analysis of evolutionary adaptation of mammalian hibernation.

Differential gene expression plays a key role in the evolution of morphological phenotypes and some biological traits [54]. The evolution of phenotypic fitness is influenced, in part, by the divergent pattern of TF binding sites and different affinities of TFs to their binding sites. The crosstalk among TFs regulates transcription, ultimately leading to the complex phenotypes [54, 55]. It has been shown that TF binding sites evolve rapidly in order to adapt to metabolic control and detoxification in the liver [56]. Our observations of more PPARα target genes and a higher binding affinity of PPARα to its target genes in hibernating mammals suggest an important regulatory role of PPARα in hibernation. It is probable that the genomes of hibernators have evolved to adapt to hibernation.

Mammalian hibernation is an ideal model to investigate the role of differential gene expression in adaptive evolution [57]. The coding regions of most differentially expressed genes are shaped by purifying selection [54]. A recent comparative genomic study of 3 hibernating and several non-hibernating species of mammals found no signs of positive selection on the coding regions of genes associated with hibernation [58]. However, differential expression of genes of metabolic pathways commonly shared by 4 hibernating species (*Myotis brandtii*, *Ursus americanus*, *Spermophilus parryii*, and *Spermophilus tridecemlineatus*) was found, indicating the pivotal role of regulation of gene expression in mammalian hibernation [57, 58]. Our findings together with previous reports [58] suggest that both genomic information (e.g., gene sequences) and genomic processes (e.g., transcription and translation) of *Ppar*α have evolved toward adaptation to hibernation. More comparative studies of TF binding between torpid and active mammals or between hibernating and non-hibernating species will allow us to gain further insights into the evolution of mammalian hibernation.

## Conclusions

In this study, we found PPARα upregulation at both mRNA and protein levels in bat liver during torpor, providing clear evidence of PPARα in adaptation to mammalian hibernation. Mechanisms by which bats upregulate the transcription and translation of PPARα to achieve a torpid phenotype remain to be investigated. A complex crosstalk among different TFs, such as FOXL1, NFYA, NFYB, SP1, TBP, ERG, RELA, and E2F1, may be involved in the control of PPARα expression. Results of evolutionary analyses and amino acid sequence alignments indicated that PPARα is highly conserved among hibernators. Bioinformatic analyses revealed that the genomes of mammalian hibernators have evolved to become more susceptible to PPARα regulation.

## Methods

### Animals and tissue acquisition

Acquisition of bats and experiments involving animals were done in accordance with ethical principles of the Animal Ethics Committee, East China Normal University (approval number AR2012/03001). 6 males each of the following hibernating bat species were captured from various locations in China: *Rhinolophus ferrumequinum* from Guan Ma Karst Cave (40°08′N, 126°05′E) in Jilin Province; *Hipposideros armiger* from Fish Cave (30°20′N, 117°50′E) in Anhui Province; *Myotis ricketti* from 7 Star Cave (25°16′N, 110°18′E) in Guangxi Province; *Taphozous melanopogon* from Ladian County (24°41′N, 108°01′E) in Guangxi Province; *Scotophilus heathii* from Mengla County (21°27′N, 101°33′E) in Yunnan Province; and *Ia io* from Feilong Cave (24°58′N, 104°53′E) in Guizhou Province (see Additional file 3: Fig 1). 3 bats of each species were sacrificed on site while they were in torpor, and the remaining bats were scarified 48hr after arousal from torpor in the laboratory. Rectal temperatures were approximately 8-10 °C for torpid and 36-37 °C for active bats. 3 males each of non-hibernating bat species *Rousettus leschenaultii*, *Cynopterus sphinx*, and *Eonycteris spelaea* were captured from Mashan County (23°55′N, 108°26′E) in Guangxi Province, Haikou Park (20°02′N, 110°20′E) in Hainan Province, and Xishuangbanna (22°0′N, 100°47′E) in Yunnan Province, China, respectively. Non-hibernating bats captured were sacrificed in the field. Mice were purchased from Sino-British Sippr/BK Lab Animal Ltd (Shanghai, China). These animals were sacrificed by cervical dislocation, and their liver tissues were rapidly excised, snap frozen in liquid nitrogen, and then stored in a −80 °C freezer until used.

### RNA Isolation, cloning, and sequencing

Total RNA was isolated from the liver tissues using Trizol® reagent (Invitrogen, USA). A total of 5 μg RNA was reverse transcribed to cDNA using the SuperScript® III Reverse Transcriptase kit (Invitrogen, USA). Primer pairs listed in Table 2 were used to amplify the coding region of bat *Ppar*α. PCRs were carried out under the following conditions: denaturation at 95 °C for 5 min; 32 cycles of 95 °C for 30 s, 50 - 60 °C for 30 s, and 72 °C for 2 min; and a final extension at 72 °C for 10 min. The resulting DNA fragments were isolated by electrophoresis in 1 % agarose gel and then ligated into pGEM®-T Easy Vector (Promega, USA). Recombinant plasmids were transformed into *E. coli* strain TOP10 (Tiangen, China). Bacterial colonies were screened by blue-white selection, and white colonies were picked for colony PCR. 6 positive clones of each PCR product were sequenced on an ABI 3730 DNA sequencer. All sequences obtained were submitted to GenBank (see Additional file 1: Table S8).

**Table 2:** Primers for cloning of bat Pparα

Name	Sequences	Amplified species	Primer used	Product length (bp)	Length proportion
*Ppar*α-1 F	5’-CTTGAGGCTGATGAYCTGGAAA	*E. spelaea*	1 and 2	1361	96.73 %
*Ppar*α-1R	5’-TYGGGAAGAGGAAGRTGTCG	*R. leschenaultii*	1 and 2	1361	96.73 %
*Ppar*α-2 F	5’-CAAYCCACCTTTYGTCAT	*C. sphinx*	1 and 2	1361	96.73 %
*Ppar*α-2R	5’-ATRTCCCTGTAGATYTCCT	*R. ferrumequinum*	1 and 3	1362	96.80 %
*Ppar*α-3 F	5’-TGAATAAAGACGGGATGCT	*H. armiger*	1 and 3	1362	96.80 %
*Ppar*α-3R	5’-CATGTCCCTGTAGATTTCCT	*T. melanopogon*	4	1118	79.46 %
*Ppar*α-4 F	5’-CGGTGTCTTACCCTGTGGT	*S. heathii*	4	1118	79.46 %
*Ppar*α-4R	5’-CGCCTCGGTCCTCTTGAT	*M. ricketti*	4	1118	79.46 %
		*I. io*	4	1118	79.46 %

### Sources of *Ppar*α nucleotide sequences

Nucleotide sequences of *Ppar*α were obtained from the following: 56 mammalian species of Plancentalia and Marsupialia [59] (see Additional file 1: Table S8 and Additional file 3: Fig S2); 5 species of Yinpterochiroptera bats including 3 non-hibernating bat species (family Pteropodidae) *C. sphinx, E. spelaea*, and *R. leschenaultii* and 2 hibernating bat species *R. ferrumequinum* (family Rhinolophidae) and *H. armiger* (family Hipposideridae); and 7 species of hibernating Yangochiroptera bats including *T. melanopogon* (family Emballonuridae) and 6 species of family Vespertilionidae: *S. heathii, I. io*, *E. fuscus*, *M. ricketti, M. brandtii*, and *M. davidii*. These bats were chosen because their hibernation behaviors are known [9, 12]*.* The DNA sequences of *Ppar*α of *E. fuscus*, *M. brandtii*, *M. davidii* and non-bat mammals, such as *Homo sapiens* and *Mus musculus*, were downloaded from GenBank.

### Evolutionary analysis of *Ppar*α of bats

The nucleotide sequences of *Ppar*α were aligned using the software Clustal X [60]. DNA sequences were translated into amino acid sequences using the software MEGA5 [61]. The program CODEML in PAML (version 4.8) was used to estimate the ω value, which is derived from *d*_*N*_ (nonsynonymous substitution rate) divided by *d*_*S*_ (synonymous substitution rate). Species topology was constructed as previously described (Fig 1) [6, 62]. An ω value of 1, < 1, or > 1 represents neutral evolution, negative purifying selection, and positive selection, respectively. The one-ratio model analysis, which assumes an equal ω value among all branches, was first conducted to establish the null hypothesis [63]. The free ratio model, which allows ω values to vary among branches, was used to compare with the one-ratio model to test the heterogeneity of ω across the tree. The two-ratio model, which allows ω values to vary between the labeled and other branches, was also applied to each of the ancestral branches of 2 clades of hibernating bats from Yinpterochiroptera and Yangochiroptera (Table 1).

### Real-time PCR

Total RNA was extracted from the liver tissues of hibernating bats *H. armiger*, *R. ferrumequinum*, and *M. ricketti*. 6 bats of each species were used, including 3 active and 3 torpid bats. The mRNA was reverse transcribed into cDNA as described above. The primer pairs used are listed in Table 3. 100 nanogram of cDNA was used to determine the expression level of *Ppar*α. Real-time PCR was performed on an ABI Prism 7300 real-time PCR system (Applied Biosystems, UK) using the SYBR® *Premix Ex Taq*^TM^ (Tli RNaseH Plus) kit (Takara, Japan). The 2^-ΔΔ*C*T^ method was applied to normalize the level of *Ppar*α to that of *Gapdh* and to calculate the relative expression levels of *Ppar*α between torpid and active bats [64-68].

**Table 3:** Primers used in RT-PCR

Genes and bat species	Primers^a^
*Ppar*α and *M. ricketti*	F: 5’-AAAGCGAAACTGAAAGCAGAAATCC
	R: 5’-TCATGTTGAAGCTCCGCAGGTAG
*Ppar*α and *R. ferrumequinum*	F: 5’-AGCCAACAACAATCCACCTTT
	R: 5’-AGCTCCGTGACAGTCTCCACA
*Ppar*α and *H. armiger*	F: 5’-TTTCACAAGTGCCTTTCGGTTGG
	R: 5’-GATTTGAGGTCCGCCGTTTCG
*Gapdh* (internal control for all bat species)	F: 5’-ATGGGTGTGAACCAYGASAAGT
	R: 5’-GGTCATGAGTCCYTCCACRAT

^a^Y = C + T, S = G + C, R = A + G.

### Western blotting

Liver tissues (100 mg) from bats and mice were homogenized separately in 2-ml Eppendorf tubes, each containing 1.7 ml of lysis buffer (10 % glycerol, 2 % SDS, 1.25 % β-mercaptoethanol, 25 mM Tris–HCl, pH 6.8) and ceramic beads (0.17 g, 1 mm in diameter), using a Precellys® 24 grinder (Bertin Technologies, France) at 4 °C. The homogenates were centrifuged at 13,400 xg for 10 min at 4 °C. Supernatants were boiled at 100 °C for 10 min and then centrifuged at 13,400 xg for 10 min at 4 °C to remove insoluble cell debris. Each of the clarified supernatants was divided into small aliquots and stored at −80 °C until used. Protein concentration of each sample was determined using the Quick Start^TM^ Bradford protein assay kit (Bio-Rad, USA) according to manufacturer’s instructions.

Equal amount of each protein sample (20 μg/lane) was subjected to a 12.5 % SDS-PAGE, and the separated proteins were transferred onto a PVDF membrane (Millipore, USA). The membrane was immersed in blocking buffer (5 % skim milk and 1 % BSA) at 4 °C overnight, and then probed with anti-PPARα (1:100 dilution, Santa Cruz®, USA, sc-9000), which is a rabbit polyclonal antibody against a conserved N-terminal epitope of PPARα. After washing with TBST buffer, the blot was reacted with an HRP-conjugated secondary antibody (Santa Cruz®, CA) at room temperature for 2 hours, followed by incubation with Immobilon^TM^ Western HRP Substrate Reagent (Millipore, USA). A reversible Ponceau S staining of the membrane was carried out to estimate the relative amount of each protein on the membrane [69]. Band intensity on the blot was quantified by the ImageQuant^TM^ TL software (version 7.0, Amersham Biosciences), and the intensity of each band was normalized to the relative value of the corresponding Ponceau-stained protein band.

### Bioinformatic Analyses of Target Genes and Determination of Affinity Scores of PPARα

The ChIP-seq data derived from chromatin immunoprecipitation (ChIP) of human hepatoma cells with anti-PPARα antibody were obtained from the GEO database (GSM864671) [36] and mapped to the mouse genome sequence (version mm9) [70]. The number of ChIP-seq peaks located in introns, exons, upstream regions of TSS (transcription start site), downstream regions of TTS, and other regions of all annotated genes in the mouse genome was separately calculated and divided by the total peak number to obtain the peak ratio of a particular location in the genome (see Additional file 3: Fig S7). A PPARα target gene was defined if at least one binding site was located within introns, exons, or 10 kb upstream of TSS of the gene.

The nucleotide sequences of 10 kb upstream of TSS, introns, and exons of all annotated genes of each of the 64 mammalian species (see Additional file 1: Table S6) were retrieved from NCBI, Ensembl, or GigaDB [71]. The position frequency matrix (PFM) of PPARα (f2) (see Additional file 3: Fig S8), which represents DNA sequence patterns recognized by PPARα, was obtained from HOCOMOCO [72]. The matrix scan was first applied to estimate the number of putative binding sites of PPARα in these sequences using parameters ‘-pseudo 1 -decimals 1 -2str -origin end -bg_pseudo 0.01 -return limits -return pval -uth pval 0.0000005’ [73]. The number of PPARα target and non-target genes of each species was calculated based on the results of matrix scan. The ratio of PPARα target gene was derived from the number of target genes divided by that of total genes (see Additional file 4). The downloaded sequences were scanned by f2 PMF using a modified SPeaker algorithm [74] to calculate the affinity score of PPARα binding to each target gene. Only the highest score estimated for a gene was adopted. Results were analyzed by two-tailed Student’s *t*-test. A *P* value < 0.05 was considered significant.

To correlate the mammalian hibernation trait with the ratio of PPARα target gene or PPARα affinity to its target genes, 2 different phylogenetic comparison methods, including the quantitative genetic threshold model [52] and the phylogenetic ANOVA [53] were used (see Additional file 1: Table S7, Additional file 2, and Additional file 3: Fig S6). The phylANOVA and threshBayes in the R package phytools were conducted separately to determine the correlations [75].

### Availability of supporting data

The data sets supporting the results of this article are available in the Dryad digital repository, [http://dx.doi.org/10.5061/dryad.h6r74] [76] DOI:10.5061/dryad.h6r74%5d%20%5b76. Gene sequences obtained in this study have been deposited in GenBank [http://www.ncbi.nlm.nih.gov/genbank]. Accession numbers are provided in Additional file 1.

## Additional files

Additional file 1: Table S1. Branch-site model A test for detection of positively selected sites in ancestral branches of hibernating bats. Table S2. Site model comparisons of 12 bat species. Table S3. Branch model tests of *Ppar*α in 56 mammalian species. Table S4. Site model comparisons of 25 mammals. Table S5. Prediction of transcription factor binding to 3 potential regulatory regions (TSS_Around, TSS_Up, and TSS_Body) of *Ppar*α*.* Table S6. Affinity scores of PPARα binding to all annotated genes in 64 mammalian species. Table S7. Phylogenetic comparative methods for determining correlations among traits. Table S8. Accession numbers of *Ppar*α sequences of 56 mammals.

Additional file 2: **Supplementary methods for evolutionary analyses of** ***Ppar*** **α in mammals and analyses of the binding affinity of each of the 205 transcription factors to the 3 potential regulatory regions of** ***Ppar*** **α in various mammalian species.**

Additional file 3: Figure S1. Geographical locations of bats obtained in China. The degrees of latitude and longitude are shown. Non-hibernating bats in Yinpterochiroptera, hibernating bats in Yinpterochiroptera, and hibernating bats in Yangochiroptera are represented by orange, green, and blue colors, respectively. Figure S2. Red dots A, B, C, and D denote the ancestral branch of Chiroptera; Cetartiodactyla, Perissodactyla, and Carnivora in Laurasiatheria; Rodentia and Lagomorpha; Scandentia and Primates in Euarchontoglires, respectively. The N*d_*N*_/S*d_*S*_/ω values of A, B, C, and D are shown below the red dots. Abbreviations Af, Au, Am, and M represent Afrotheria, Australidelphia, Ameridelphia, and Marsupialia, respectively. Red asterisks indicate the species that can hibernate. Figure S3. Amino acid sequence alignments of PPARα of bats. Amino acid sequences of non-hibernating and hibernating bat species are denoted with pink and blue colors, respectively. The amino acid positions that are conserved in non-hibernating species but different or diverged among hibernating species are indicated with orange triangles. Amino acid sites that are conserved in hibernating species but different or diverged among non-hibernating species are denoted with red triangles. The green line shows the location of the zinc-finger motif. Figure S4. (A) The ratio of the number of PPARα potential target genes to that of all genes in bats. The small open rectangle indicates the threshold setting for the matrix scan. (B) Mean scores of binding affinity of PPARα to its target genes in bats. Blue and pink dots represent the mean value calculated from hibernating and non-hibernating bats, respectively. Figure S5. The y-axis in (A) represents the ratios of the number of PPARα potential target genes to the number of all genes of different animals. The small open rectangle indicates the threshold setting for the matrix scan. The scores in (B) indicate the binding affinity of PPARα to its target genes in different animals. Blue and pink boxes indicate data calculated from 16 hibernating species (H) and 48 non-hibernating species (N) of mammals. Each dot represents the mean value obtained from each mammalian species. The box plot shows the median, 25/75 percentiles (box), 10/90 percentiles (bars). A *P* value < 0.05 is considered significant. Figure S6. (A) Phylogenetic tree constructed for 62 mammalian species. Hibernating species are colored in red. (B) Results of phylogenetic ANOVA. The y-axis denotes scores of binding affinity of PPARα to its target genes. The box plot shows the median, 25/75 percentiles (box), and 10/90 percentiles (bars). A *P* value < 0.05 is considered significant. Figure S7. Mapping of PPARα binding sites to the mouse genome. The y-axis denotes the number of PPARα binding sites located in various regions of all annotated genes, including the sequences upstream of TSS (UpStream), 5’ un-translated sequences (5’UTR), exons (Coding Exon), introns (Intron), 3’ untranslated sequences (3’UTR), the sequences downstream of TSS (DownStream), and the distal sequences (>20 kb) (Distal_Region). The Arabic number shown on top of each bar indicates the ratio of the number of binding sites in the region to the number of all PPARα binding sites. Figure S8. Position frequency matrix (PFM) of sequences recognized by PPARα.

Additional file 4: **Different threshold settings of** ***P*** **value for the matrix scan of PPARα in mammals.**

## Abbreviations

PPARα: Peroxisome proliferator-activated receptor alpha
RXR: Retinoid X receptor
TFs: Transcription factors
FOXL1: Forkhead box protein L1
NFYA: Nuclear transcription factor Y subunit alpha
NFYB: Nuclear transcription factor Y subunit beta SP1, Specificity protein 1
TBP: TATA-box-binding protein 1
ERG: E-26 related gene
RELA: v-rel avian reticuloendotheliosis viral oncogene homolog A
E2F1: E2 Transcription Factor 1
