# Supplementary material for: Adaptation of peroxisome proliferator-activated receptor alpha to hibernation in bats
Source: BMC Evol Biol. 2015 May 17;15:88. doi: 10.1186/s12862-015-0373-6 (PMC4435907; doi:10.1186/s12862-015-0373-6)
Supplement: Supplementary file 2 — Supplementary methods for evolutionary analyses of Ppar α in mammals and analyses of the binding affinity of each of the 205 transcription factors to the 3 potential regulatory regions of Ppar α in various mammalian species. [file 12862_2015_373_MOESM2_ESM.pdf]

Additional file 2: Supplementary Methods

Evolution Analyses of *Ppara* of bats

The branch-site model A, which allows ω to vary among sites in *Ppara* and across branches on the tree (Figure 1), was applied to detect positive selections on sites along particular lineages (see Additional file 1: Table S1) [1]. The phylogeny was divided into foreground and background branches in this model. Four site classes of codons were assumed. Classes 0 and 1 evolved under purifying selection ($0 < \omega_0 < 1$) and neutral selection ($\omega_1 = 1$), respectively, throughout the tree. Classes 2a and 2b evolved under purifying ($0 < \omega_0 < 1$) and neutral selection ($\omega_1 = 1$) on the background, respectively. They were grouped together and allowed to evolve under positive selection on foreground ($\omega_2 > 1$). Both tests 1 and 2 of the branch-site model [2] were applied to the ancestral branches of hibernating bats in Yinpterochiroptera and Yangochiroptera. In these tests, the branch-site model A was the alternative hypothesis. The M1a (nearly neutral) model assumed two site classes: $0 < \omega_0 < 1$ and $\omega_1 = 1$. The modified branch-site model A had ω_2 fixed as 1. M1a served as the null hypothesis for test 1, and the modified branch-site model A served as the null hypothesis for test 2. All results of alternative and null hypotheses were compared using the likelihood ratio test. The site model tests were conducted by three pairs of models: M2a (positive selection) versus M1a (nearly neutral), M8 (β & ω) versus M7 (β), and M3 (discrete) versus M0 (one ratio) (see Additional file 1: Table S2) [3].

Evolution Analyses of *Ppara* of mammals

Nucleotide sequences of *Ppara* from various mammalian species were aligned using the software Clustal X [4]. Species topology was constructed as previously described (see Additional file 3: Figure S2) [5, 6]. The program CODEML in PAML (version 4.8) was used to estimate the ω value, which is derived from d_N (nonsynonymous substitution rate) divided by d_S (synonymous substitution rate) (see Additional file 1: Table S3) [7]. An ω value of 1, < 1, or > 1 represents neutral evolution, negative purifying selection, and positive selection, respectively. The one-ratio model analysis, which assumes an equal ω value among all branches, was first conducted to establish

the null hypothesis. The free ratio model, which allows ω values to vary among branches, was used to compare with the one-ratio model to test the heterogeneity of ω across the species tree. The two-ratio model, which allows ω values to vary between the labeled and other branches, was also applied to each of the ancestral branch of Laurasiatheria, Euarchontoglires, and Chiroptera. The amino acid sites that are under positive selection were identified by three pairs of models: M2a (positive selection) versus M1a (nearly neutral), M8 (β & ω) versus M7 (β), and M3 (discrete) versus M0 (one ratio) (see Additional file 1: Table S4) [3].

Affinity Analyses of 205 TFs to *Ppara*

The position frequency matrices (PFMs) of 205 transcription factors (TFs) for DNA recognition were downloaded from the Jaspar database [8]. The nucleotide sequences of upstream 10 kb from TSS, introns, and exons of *Ppara* of sixty-four mammalian species (forty-eight hibernating and sixteen non-hibernating species) were retrieved from NCBI or Ensembl (see Additional file 1: Table S6). These sequences were separately scanned by PFMs using a modified SPeaker algorithm [9] to calculate the affinity scores of these TFs to *Ppara*. Only the highest score of a TF was adopted (see Additional file 1: Table S5).

Phylogenetic Approaches in Comparative Physiology

The species topology of sixty-four mammals was constructed as previously described [6, 10-12]. Branch lengths of the phylogeny were estimated using the methods of Grafen and Garland [13-15] (see Additional file 3: Figure S6A). Phylogenetic comparisons were carried out by the threshold model [16] or phylogenetic ANOVA (see Additional file 3: Figure S6B) [17]. The threshBayes and phylANOVA in the R package phytools were conducted separately [18]. For results generated from the threshold model, the 95% confidence intervals of new correlation coefficient were estimated using smean.cl.boot in Harrell's Hmisc package {R} [19]. A result was considered significant when the confidence interval was above or below zero.

References

1. Yang Z, Wong WSW, Nielsen R: **Bayes empirical Bayes inference of amino acid sites under positive selection**. *Molecular biology and evolution* 2005, **22**(4):1107-1118.

2. Zhang J, Nielsen R, Yang Z: **Evaluation of an improved branch-site likelihood method for detecting positive selection at the molecular level**. *Molecular biology and evolution* 2005, **22**(12):2472-2479.
3. Nielsen R, Yang Z: **Likelihood models for detecting positively selected amino acid sites and applications to the HIV-1 envelope gene**. *Genetics* 1998, **148**(3):929-936.
4. Larkin MA, Blackshields G, Brown N, Chenna R, McGettigan PA, McWilliam H, Valentin F, Wallace IM, Wilm A, Lopez R: **Clustal W and Clustal X version 2.0**. *Bioinformatics* 2007, **23**(21):2947-2948.
5. Teeling EC, Springer MS, Madsen O, Bates P, O'Brien SJ, Murphy WJ: **A molecular phylogeny for bats illuminates biogeography and the fossil record**. *Science* 2005, **307**(5709):580-584.
6. Zhou X, Xu S, Xu J, Chen B, Zhou K, Yang G: **Phylogenomic analysis resolves the interordinal relationships and rapid diversification of the Laurasiatherian mammals**. *Systematic biology* 2012, **61**(1):150-164.
7. Yang Z: **PAML 4: phylogenetic analysis by maximum likelihood**. *Molecular biology and evolution* 2007, **24**(8):1586-1591.
8. Wasserman WW, Sandelin A: **Applied bioinformatics for the identification of regulatory elements**. *Nature Reviews Genetics* 2004, **5**(4):276-287.
9. Megraw M, Pereira F, Jensen ST, Ohler U, Hatzigeorgiou AG: **A transcription factor affinity-based code for mammalian transcription initiation**. *Genome research* 2009, **19**(4):644-656.
10. Murphy WJ, Pevzner PA, O'Brien SJ: **Mammalian phylogenomics comes of age**. *Trends in Genetics* 2004, **20**(12):631-639.
11. Steppan SJ, Adkins RM, Anderson J: **Phylogeny and divergence-date estimates of rapid radiations in muroid rodents based on multiple nuclear genes**. *Systematic Biology* 2004, **53**(4):533-553.
12. Álvarez A, Perez SI, Verzi DH: **Ecological and phylogenetic dimensions of cranial shape diversification in South American caviomorph rodents (Rodentia: Hystricomorpha)**. *Biological Journal of the Linnean Society* 2013, **110**(4):898-913.
13. Grafen A: **The phylogenetic regression**. *Philosophical Transactions of the Royal Society of London Series B, Biological Sciences* 1989:119-157.
14. Garland T, Harvey PH, Ives AR: **Procedures for the analysis of comparative data using phylogenetically independent contrasts**. *Systematic Biology* 1992, **41**(1):18-32.
15. Garland T, Diaz-Uriarte R: **Polytomies and phylogenetically independent contrasts: examination of the bounded degrees of freedom approach**. *Systematic Biology* 1999, **48**(3):547-558.
16. Felsenstein J: **A comparative method for both discrete and continuous characters using the threshold model**. *The American Naturalist* 2012, **179**(2):145-156.
17. Garland T, Dickerman AW, Janis CM, Jones JA: **Phylogenetic analysis of covariance by computer simulation**. *Systematic Biology* 1993, **42**(3):265-292.
18. Revell LJ: **Phylogenetic Tools for comparative biology (and other things)**. [http://www.phytools.org]
19. Harrell FE, Dupont C: **Hmisc: Harrell miscellaneous. R package version 3.9-3**. [http://CRAN.R-project.org/package=rms]
