# Supplementary figures and images for: Adaptation of peroxisome proliferator-activated receptor alpha to hibernation in bats
Source: BMC Evol Biol. 2015 May 17;15:88. doi: 10.1186/s12862-015-0373-6 (PMC4435907; doi:10.1186/s12862-015-0373-6)

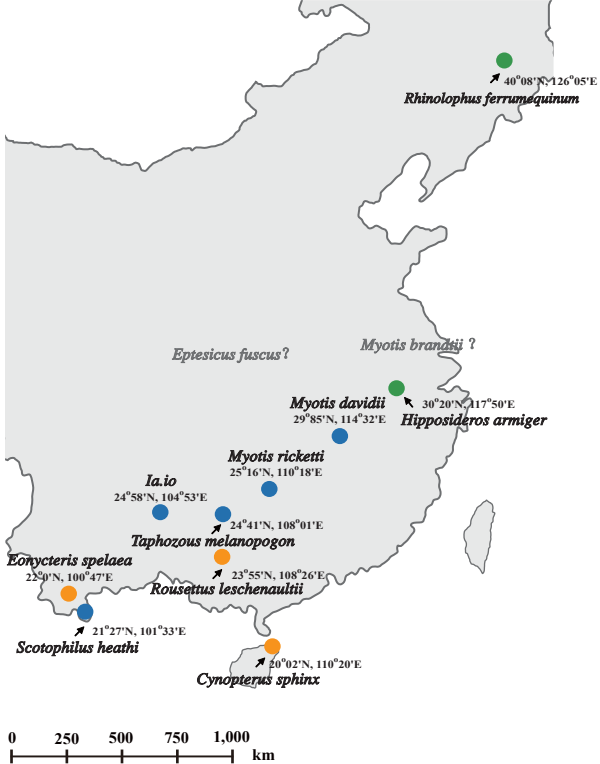

Supplement: Supplementary file 3 — Geographical locations of bats obtained in China. The degrees of latitude and longitude are shown. Non-hibernating bats in Yinpterochiroptera, hibernating bats in Yinpterochiroptera, and hibernating bats in Yangochiroptera are represented by orange, green, and blue colors, respectively. Figure S2. Red dots A, B, C, and D denote the ancestral branch of Chiroptera; Cetartiodactyla, Perissodactyla, and Carnivora in Laurasiatheria; Rodentia and Lagomorpha; Scandentia and Primates in Euarchontoglires, respectively. The N*d N/S*d S/ω values of A, B, C, and D are shown below the red dots. Abbreviations Af, Au, Am, and M represent Afrotheria, Australidelphia, Ameridelphia, and Marsupialia, respectively. Red asterisks indicate the species that can hibernate. Figure S3. Amino acid sequence alignments of PPARα of bats. Amino acid sequences of non-hibernating and hibernating bat species are denoted with pink and blue colors, respectively. The amino acid positions that are conserved in non-hibernating species but different or diverged among hibernating species are indicated with orange triangles. Amino acid sites that are conserved in hibernating species but different or diverged among non-hibernating species are denoted with red triangles. The green line shows the location of the zinc-finger motif. Figure S4. (A) The ratio of the number of PPARα potential target genes to that of all genes in bats. The small open rectangle indicates the threshold setting for the matrix scan. (B) Mean scores of binding affinity of PPARα to its target genes in bats. Blue and pink dots represent the mean value calculated from hibernating and non-hibernating bats, respectively. Figure S5. The y-axis in (A) represents the ratios of the number of PPARα potential target genes to the number of all genes of different animals. The small open rectangle indicates the threshold setting for the matrix scan. The scores in (B) indicate the binding affinity of PPARα to its target genes in different animals. Blue [file 12862_2015_373_MOESM3_ESM.pdf]
